# Recurrent Henoch-Schönlein Purpura with bullous rash and pulmonary nodules

**DOI:** 10.1186/s12969-020-00436-7

**Published:** 2020-05-24

**Authors:** Christopher Zheng, Julie Childers, Egla Rabinovich, Kristina Nazareth-Pidgeon

**Affiliations:** Department of Pediatrics, Duke University Hospital, 2301 Erwin Rd, Durham, NC 27705 USA

**Keywords:** Henoch-Schönlein purpura, Bullous rash., Pulmonary nodules.

## Abstract

**Background:**

Henoch-Schönlein purpura (HSP) is the most common vasculitis of childhood. It has a characteristic rash described as palpable purpura that most frequently affects the distal lower extremities and buttocks. HSP rarely presents with bullous rash nor pulmonary nodules.

**Case presentation:**

We present a novel case of a 12-years-old female with recurrent pediatric HSP with a combination of the rare manifestations of bullous rash and pulmonary nodules. She initially presented with the bullous rash, chest pain, cough, and abdominal pain. Patient was successfully treated with intravenous pulse corticosteroids followed by a high dose oral corticosteroid taper, with resolution of the bullous rash and pulmonary nodules.

**Conclusion:**

The rare manifestations of scarring bullous rash and pulmonary nodules can be presenting features of pediatric HSP, the combination of which has not been previously reported. The treatment of intravenous corticosteroid resolved patient’s abdominal symptoms, rash and pulmonary nodules.

## Background

Henoch-Schönlein Purpura is an IgA-mediated leukocytoclastic vasculitis involving small vessels. It affects approximately 10–20 children per 100,000 each year [1], with recurrence in approximately one-fifth to one-fourth of cases [2, 3]. The characteristic initial presentation of palpable purpura with a lower extremity predominance is typically followed by gastrointestinal manifestations, renal involvement, and/or joint pain.

While not common, HSP can also present with bullous rash, with an estimated prevalence of less than 2% in pediatric HSP patients [4, 5]. Similarly rare, the prevalence of HSP with pulmonary involvement is approximately 0.8 to 5% [6]. The combination of both bullous rash and pulmonary nodules in a single patient diagnosed with HSP has not been reported in the literature to date. Treatment with corticosteroid is effective in HSP patients with these atypical manifestations [4, 6].

## Case presentation

**Fig. 1:**
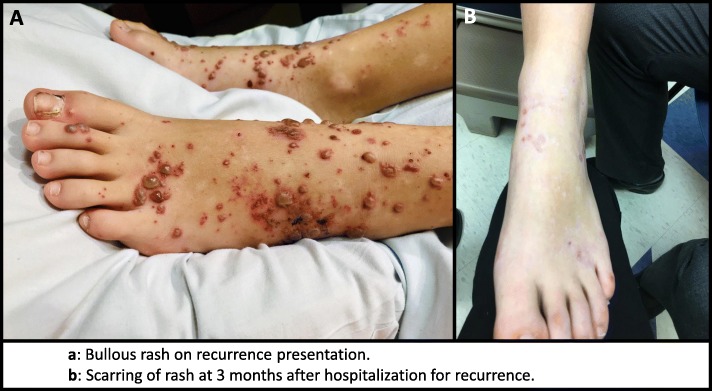
**a**. Bullous rash on recurrence presentation. **b**. Scarring of rash at 3 months after hospitalization for recurrence

A 12-year-old girl presented in early October with a one-week history of progressive and painful bullous rash of the distal lower extremities (Fig. 1a), abdominal pain, nonproductive cough, and pleuritic chest pain. She denied fevers, mucosal lesions, hematemesis, arthralgia, hematuria, melena, or hematochezia. Her medical history was notable for an identical rash that had been diagnosed as HSP four years prior, accompanied by significant abdominal pain, hematuria, and proteinuria; there was no pulmonary involvement noted at that time. She had been treated with a high-dose oral prednisone taper but did not show improvement until several weeks following completion of that treatment course. She was subsequently symptom-free in the interim. She traveled to the Outer Banks of North Carolina in the months preceding her presentation but had no other travel history.

On physical exam, the rash on her feet and ankles was bullous and in various stages of healing, with exquisite tenderness to palpation and positive Nikolsky’s sign. She had clustered vesicles with erythematous base on the bilateral extensor surfaces of her elbows as well as scattered non-blanching pinpoint erythematous macules at the inferior aspect of her bilateral buttocks. Cardiopulmonary exam was unremarkable except for an intermittent, dry cough. Her chest pain was not reproducible with palpation.

**Fig. 2:**
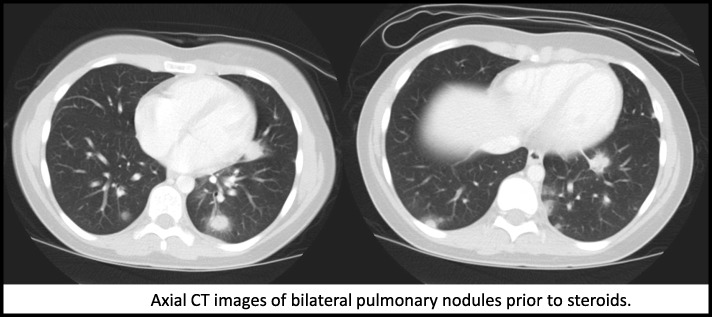
Axial CT images of bilateral pulmonary nodules prior to steroids

Basic labs were remarkable for elevations in inflammatory markers and a mildly impaired prothrombin time. Complete blood count and comprehensive metabolic panel were otherwise normal. Urinalysis revealed microscopic hematuria and mildly elevated protein-to-creatinine ratio. Chest X-ray was concerning for pulmonary infiltrates. Computed tomography (CT) of the chest revealed multiple peripheral pulmonary nodules concerning for a possible fungal infection versus hemorrhagic vasculitic process (Fig. 2). Abdominal ultrasounds throughout the admission were negative for intussusception, a known complication of HSP resulting from submucosal hematoma or edema.

Further workup revealed the following:

### Skin

**Fig. 3:**
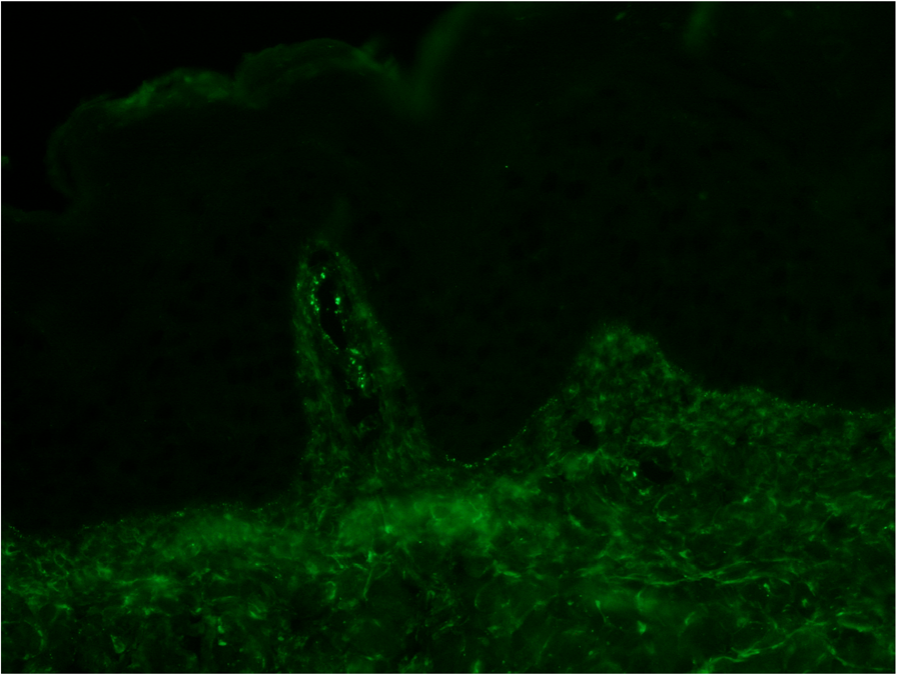
C3 direct immunofluorescent staining

**Fig. 4:**
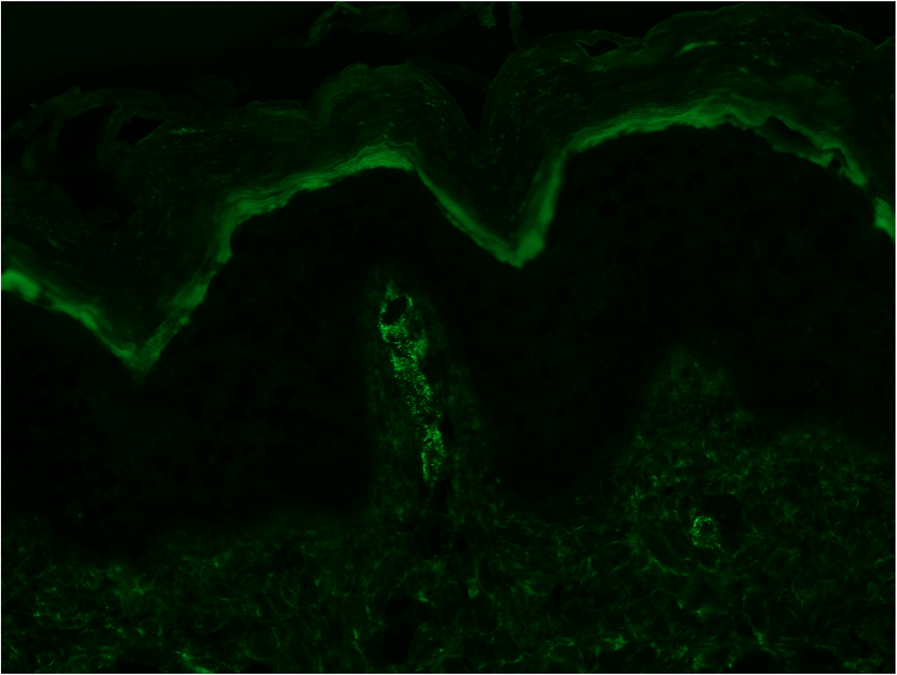
IgA direct immunofluorescent staining

**Fig. 5:**
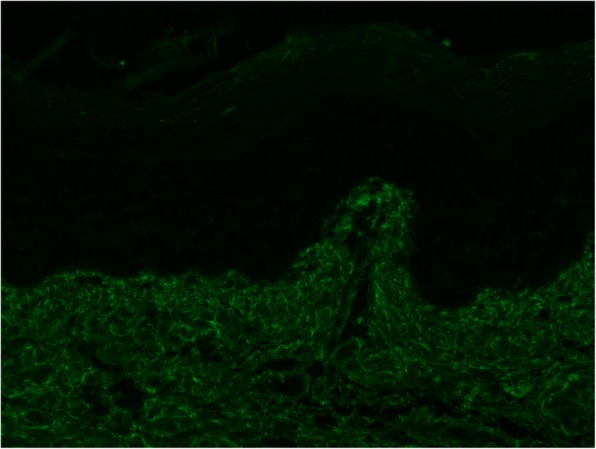
Negative IgG direct immunofluorescent staining

**Fig. 6:**
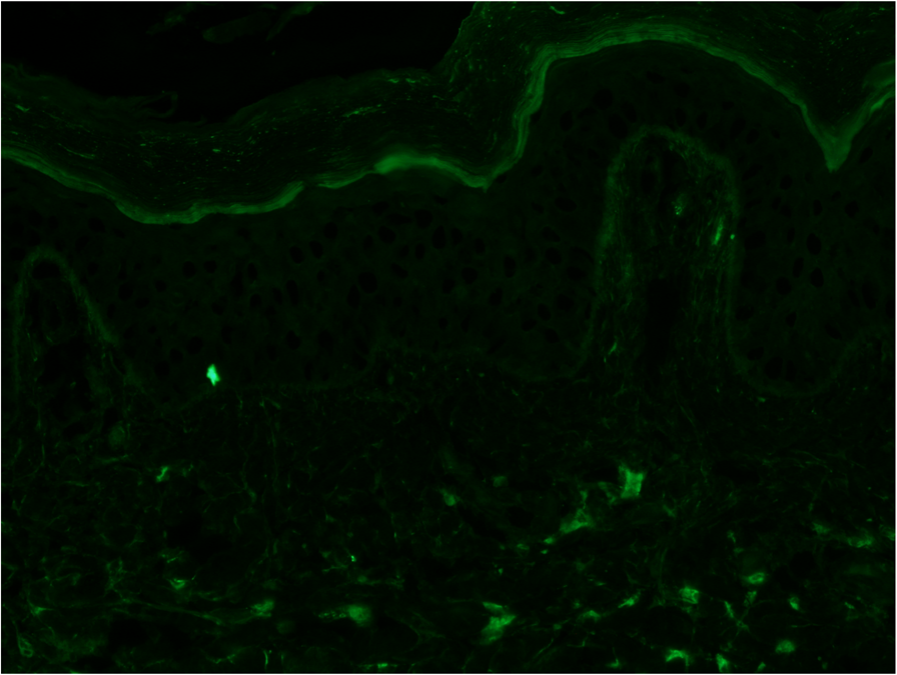
Negative IgM direct immunofluorescent staining

The patient’s skin lesions were biopsied. Microscopic examination was consistent with leukocytoclastic vasculitis. Direct immunofluorescence revealed granular IgA (Fig. 3), C3 (Fig. 4), and fibrin deposits around scattered blood vessels in the upper dermis; no diagnostic IgG (Fig. 5) or IgM (Fig. 6) deposits were found.

### Serum & Urine

Urinalysis showed 8 red blood cells under microscopy. Random urine protein was 39 mg/dL with a urine protein-to-creatinine ratio of 207 mg/g. Urine histoplasmosis and blastomyces antigens, and serum aspergillus antigen returned negative. Serum IgA was normal with negative tissue transglutaminase (TTG) and endomysial antibodies. Her ESR was 42 mm/hr., and CRP was 2.69 mg/dL. Serum ANCA, ANA, ASO/anti-DNase B, and anti-glomerular basement membrane antibodies were all negative. Quantiferon Gold was negative.

### Pulmonary

The patient underwent a bronchoalveolar lavage (BAL) and CT-guided fine needle aspiration (FNA) of the peripheral pulmonary nodules. BAL samples revealed normal cell counts, negative fungitell assay, negative bacterial and fungal stains and cultures, negative histoplasma and blastomyces PCR, and absence of hemosiderin-laden macrophages. The pulmonary nodule FNA had a negative fungal stain and culture, as well as negative on acid-fast bacilli smear and culture; no granulomas were seen. Cytology of both BAL and FNA revealed benign bronchial cells. The FNA samples were insufficient for evaluation of vasculitis.

The results of our investigation supported the diagnosis of recurrent bullous HSP with pulmonary nodules as a new associated element of her disease flare. The mainstay of symptomatic pain management centered around the neuropathic pain of her bilateral feet and ankles (described as burning and shooting in nature); patient was given scheduled gabapentin, rapidly up-titrated to a 700 mg total daily dose by hospital day 4 with near total pain resolution. Both the pleuritic chest pain and abdominal pain persisted and were managed with as-needed oral or intravenous analgesia that provided minor benefit.

On day 6 of hospitalization, after blood cultures and a pulmonary nodule biopsy were confirmed negative for infection, intravenous methlyprednisolone (10 mg/kg/day for three days) was initiated. Her abdominal pain resolved by the following day, and the pleuritic chest pain resolved shortly thereafter. Chest X-rays following her corticosteroid burst showed interval improvement in the previously-noted opacities. The patient was discharged on a two-month taper of prednisone starting at 1 mg/kg daily.

Pulmonary function test (PFT) after completing the corticosteroid course showed normal spirometry and lung volumes, with mildly reduced lung diffusion; there was no baseline PFT for comparison. Surveillance for renal manifestations continued in the outpatient setting with no signs of renal complications in the six months after discharge. Outpatient urine studies showed stable protein-to-creatinine ratio of around 300 mg/g. The patient had two episodes of arthralgias in her knees and ankles while she was still on her corticosteroid taper. Her lower extremity rashes continue to heal without additional flares, albeit slowly and with scarring (Fig. 1b). ESR and CRP both normalized on recheck five weeks after discharge, with an ESR of 5 mm/hr. and CRP down to 0.02 mg/dL. Pain was well-managed with home gabapentin, and her mother noted a significant improvement in pain management compared with treatment with acetaminophen and other oral analgesics during initial flare four years ago.

## Discussion

Our patient met the clinical criteria for HSP with atypical manifestations, as supported by our extensive serologic workup and histopathologic results that are diagnostic for HSP. While the initial differential for our patient was broad, all reasonable alternative diagnoses were considered and ruled out. Possible rheumatologic causes on the differential included pauci-immune vasculitides, Goodpastures syndrome, and systemic lupus erythematous; the negative ANCA, anti-glomerular basement membrane antibodies, and ANA lowered the likelihood of these diagnoses. Polyarteritis nodosa is ruled out by the presence of IgA deposition on skin biopsy, as well as the lack of clinical findings of renal artery stenosis (no hypertension nor congruent imaging findings) and mesentery ischemia (no postprandial abdominal pain), in lieu of a kidney biopsy. The appearance of the rash also raised the possibility of dermatitis herpetiformis and linear IgA bullous dermatosis. These two differential diagnoses were ruled out by the negative TTG/endomysial antibodies and a lack of linear band of IgA at the dermoepidermal junction, respectively. Possible infectious causes included streptococcus infection, tuberculosis, and fungal infections. A lack of convincing exposure history and low suspicion for immunocompromised state made fungal infections less likely, though the planned course of corticosteroids necessitated a fungal rule out. Extensive infectious workup including urinalysis, serum studies, BAL, and pulmonary nodule FNA biopsy were negative. Thus, our patient had most likely suffered from a rare pulmonary manifestation of HSP with an atypical scarring bullous rash.

IgA immune complexes typically spare the pulmonary vasculature. Therefore, significant pulmonary disease is rarely seen in cases of pediatric HSP, with an estimated prevalence of 0.8 to 5% [6]. A common pulmonary manifestation reported in literature appears to be diffuse alveolar hemorrhage (DAH), and those patients are typically older with extensive organ involvement [6, 7]. In a recent review by Di Pietro et al., DAH is the most common pulmonary manifestation in children, with the average age of onset of 10 years old [8]. Approximately 90% of classic HSP patients are under 10 years of age [1], whereas 50% of HSP patients with DAH are older than 20 years [6]. A less common pulmonary manifestation of interstitial pneumonia has been reported in a Mayo Clinic case series [9]. To our knowledge and literature review, there is no mention of pulmonary nodules in the existing publications. A prospective study monitoring the pulmonary function of 11 pediatric patients with HSP and without initial pulmonary involvement found no significant impairments on pulmonary function tests [10] after four years.

The prevalence of HSP with bullous rash differs drastically between adults and children. Adult HSP patients with bullous rash have been reported in 16 to 60% of cases, while less than 2% of children with HSP have a bullous rash, with a mean age of diagnosis of 8.2 years [4, 5]. Other less common skin manifestations of HSP include targetoid lesions, subcutaneous nodules, and vesicular lesions; the last of which was also noted on our patient. Similar to the classic purpuric rash, the bullous rash has a predilection for the lower extremity [4]. There does not appear to be any clear prognostic indication associated with the bullous rash [4]. Most bullous rashes resolve completely, with one case review reporting residual scars in 6 out of 38 patients [5].

The pharmacologic treatment of HSP focuses on immunosuppression, as the mechanism of tissue damage is secondary to IgA deposition and leukocyte infiltration. Findings from a 2007 meta-analysis suggest that early corticosteroid administration decreases abdominal pain duration and incidence of intussusception; there is conflicting data on whether corticosteroids can decrease the chance of HSP recurrence [11]. Irrespective of the pharmacologic therapy of choice, two retrospective studies published in 2010 and 2018 reported HSP recurrence rate of 16.4% (*n* = 1002) [2] and 23.3% (*n* = 425) [3], respectively. Aside from corticosteroids, several other immunosuppressants have been reported in the treatment of HSP with severe abdominal and renal manifestations, including azathioprine, cyclophosphamide, IVIG, plasma exchange, dapsone, and colchicine [12–14]. In HSP patients with DAH, effective treatments include pulse corticosteroids with azathioprine, cyclophosphamide, or cyclosporine [6, 7]. A recent review article by Di Pietro et al. notes the lack of standard therapy for patient with HSP with pulmonary involvement. They suggest that the first line should be intravenous methylprednisolone, followed by azathioprine or cyclophosphamide in the setting of respiratory failure [8].

The treatment of bullous HSP, per the available case reports, mainly involves corticosteroids [4, 5, 14]. Our patient had received two different corticosteroid courses, on initial presentation years prior and upon recurrence. During her first episode of bullous HSP, she received a high-dose course of 2 mg/kg oral prednisolone followed by a 3-week taper. This led to no significant improvements in her rash or abdominal pain. It has been suggested that patients with moderate disease respond much better to intravenous dosing of corticosteroids, which may be due to impaired gut absorption with vasculitis [3]. Upon disease recurrence, our patient received intravenous methylprednisolone 10 mg/kg/day for three days, then transitioned to oral prednisone 1 mg/kg/day tapered over two months. Her abdominal pain had resolved by the end of the pulse corticosteroid course. Her rash healed without further flares, albeit with significant scarring. Her pulmonary nodules without DAH improved on repeat chest radiographs. Had our patient not responded to the corticosteroids, we would have started an immunosuppressant. Based on our patient’s clinical response to corticosteroids on recurrence, we question whether oral formulations of corticosteroid may be poorly absorbed in patients with severe abdominal HSP vasculitis and thus less effective.

## Conclusion

Individually, both scarring bullous rash and pulmonary nodules are rare manifestations of pediatric HSP; we have not encountered this combination of HSP symptoms in the published literature. Our patient’s initial presentation was inadequately treated with oral high dose corticosteroids (2 mg/kg/day) plus taper, while the recurrence was effectively managed with intravenous pulse corticosteroids (10 mg/kg/day) followed by prolonged taper. Our patient received clear clinical benefit from corticosteroid therapy in resolving her abdominal symptoms, rash, and pulmonary nodules; these results align with conclusions made by Weiss et al. in their 2007 meta-analysis [11]. Their statistical analysis also suggested but failed to identify a significant corticosteroid dose-response effect [11]. Our case thus supports the use of intravenous pulse corticosteroids for the management of recurrent pediatric HSP with atypical presentations of bullous rash and pulmonary nodules.

## Data Availability

Not applicable.

## Abbreviations

HSP: Henoch-Schönlein Purpura
CT: Computed tomography
TTG: Tissue transglutaminase
BAL: Bronchoalveolar lavage
FNA: Fine needle aspiration
DAH: Diffuse alveolar hemorrhage
PFT: Pulmonary function test

## References

[1] Hetland LE, Susrud KS, Lindahl KH, Bygum A (2017). Henoch-Schonlein Purpura: a literature review. Acta Derm Venereol.

[2] Lei WT, Tsai PL, Chu SH, Kao YH, Lin CY, Fang LC (2018). Incidence and risk factors for recurrent Henoch-Schonlein purpura in children from a 16-year nationwide database. Pediatr Rheumatol Online J..

[3] Deng F, Lu L, Zhang Q, Hu B, Wang SJ, Huang N (2010). Henoch-Schonlein purpura in childhood: treatment and prognosis. Analysis of 425 cases over a 5-year period. Clin Rheumatol.

[4] Trapani S, Mariotti P, Resti M, Nappini L, de Martino M, Falcini F (2010). Severe hemorrhagic bullous lesions in Henoch Schonlein purpura: three pediatric cases and review of the literature. Rheumatol Int.

[5] Su HW, Chen CY, Chiou YH (2018). Hemorrhagic bullous lesions in Henoch-Schonlein purpura: a case report and review of the literature. BMC Pediatr.

[6] Rajagopala S, Shobha V, Devaraj U, D'Souza G, Garg I (2013). Pulmonary hemorrhage in Henoch-Schonlein purpura: case report and systematic review of the english literature. Semin Arthritis Rheum.

[7] Chen SY, Chang KC, Yu MC, Asueh S, Ou LS (2011). Pulmonary hemorrhage associated with Henoch-Schonlein purpura in pediatric patients: case report and review of the literature. Semin Arthritis Rheum.

[8] Di Pietro GM, Castellazzi ML, Mastrangelo A, Montini G, Marchisio P, Tagliabue C (2019). Henoch-Schonlein Purpura in children: not only kidney but also lung. Pediatr Rheumatol Online J.

[9] Nadrous HF, Yu AC, Specks U, Ryu JH (2004). Pulmonary involvement in Henoch-Schonlein purpura. Mayo Clin Proc.

[10] Grabska-Kobylecka I, Nowak D, Wlodarczyk A, Bialasiewicz P (2016). No impairment of pulmonary function in children with Henoch-Schonlein purpura after 4-year follow-up. Clin Rheumatol.

[11] Weiss PF, Feinstein JA, Luan X, Burnham JM, Feudtner C (2007). Effects of corticosteroid on Henoch-Schonlein purpura: a systematic review. Pediatrics.

[12] Kang HS, Chung HS, Kang KS, Han KH (2015). High-dose methylprednisolone pulse therapy for treatment of refractory intestinal involvement caused by Henoch-Schonlein purpura: a case report. J Med Case Rep.

[13] Allali S, Fraitag S, Terrier B, Bodemer C, Chalumeau M (2016). Efficacy of colchicine in a child with relapsing bullous Henoch-Schonlein purpura. Eur J Pediatr.

[14] Kausar S, Yalamanchili A (2009). Management of haemorrhagic bullous lesions in Henoch-Schonlein purpura: is there any consensus?. J Dermatolog Treat.

